# Development of Meaningful Vocal Signals in a Juvenile Territorial Songbird (*Gymnorhina tibicen*) and the Dilemma of Vocal Taboos Concerning Neighbours and Strangers

**DOI:** 10.3390/ani8120228

**Published:** 2018-11-30

**Authors:** Gisela Kaplan

**Affiliations:** School of Science and Technology, University of New England, Armidale, NSW 2351, Australia; gkaplan@une.edu.au

**Keywords:** territoriality, juvenile vocal development, neighbour–stranger discrimination, Australian magpies

## Abstract

**Simple Summary:**

Ownership of a territory is very important for many vertebrates. Some songbird species, such as the Australian magpie, have all-year-round, not just seasonal, territories. Hence, there is a good deal at stake in holding on to such a territory. It requires vigilance, experience, an excellent memory, and most of all, effective vocal communication. There is a strong relationship between successful territorial ownership, strong social bonds, and the health, survival, even the life-span and overall cognitive development of offspring. Yet, we know next to nothing as to how juveniles acquire their vocal skills and apply these appropriately, including territorial signals. Clearly, juveniles need to learn about territoriality, i.e., learn about potential enemies, recognise which birds are neighbours, and what constitutes an incursion into territory. The results of this extensive field study unexpectedly revealed that juveniles did not use more than half of all the vocalisations adults expressed and they needed a long time (three months post fledging) to distinguish between neighbour and stranger calls, attributing differential importance to each. It also showed that the territorial call was learned but was expected by adults not be used by juveniles, limiting their ability to communicate. A range of other calls associated with territoriality were also not expressed, possibly because only territory owners could do so.

**Abstract:**

Young territorial songbirds have calls to learn, especially calls that may be vital for maintaining territory. Territoriality is largely reinforced and communicated by vocal signals. In their natal territory, juvenile magpies (*Gymnorhina tibicen*) enjoy protection from predators for 8–9 months. It is not at all clear, however, when and how a young territorial songbird learns to distinguish the meaning of calls and songs expressed by parents, conspecifics, neighbours, and heterospecifics, or how territorial calls are incorporated into the juvenile’s own repertoire. This project investigated acquisition and expression of the vocal repertoire in juvenile magpies and assessed the responses of adults and juveniles to playbacks of neighbour and stranger calls inside their territory. The results reported here identify age of appearance of specific vocalisations and the limits of their expression in juveniles. One new and surprising result was that many types of adult vocalisation were not voiced by juveniles. Playbacks of calls of neighbours and strangers inside the natal territory further established that adults responded strongly but differentially to neighbours versus strangers. By contrast, juveniles needed months before paying any attention to and distinguishing between neighbour and stranger calls and eventually did so only in non-vocal ways (such as referral to adults). These results provide evidence that auditory perception not only includes recognition and memory of neighbour calls but also an assessment of the importance of such calls in the context of territoriality.

## 1. Introduction

**Figure 1:**
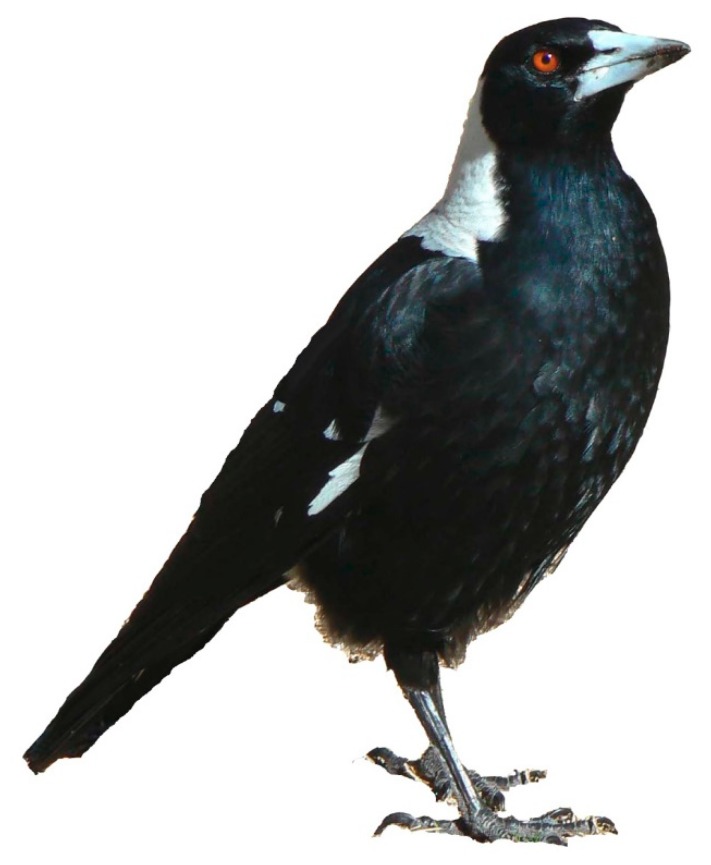
The Australian magpie is a relatively large songbird (38–46 cm), within Australia in a weight class almost on its own (except for the house crow, *Corvus splendens*). Life expectancy for those surviving the first five years without territory is about 25–30 years. Magpies form strong alliances and often life-long social pair bonds. All-year round permanent territories are defended by males and females alike. Both males and females sing, with females often having a slightly larger repertoire. Magpies are life-long learners, and once a territory has been successfully claimed and defended, they share about 25% of their repertoire with neighbours [2,3]. Males and females rear offspring jointly, but in some cases and regions, magpies also breed cooperatively [3,4,5]. Note: the Australian magpie currently goes under two different Latin names: Cracticus tibicen (subsuming tibicen under butcherbirds) and Gymnorhina tibicen (its own genus). Gymnorhina, as used here, is in accordance with the official and current IOC World Bird List [6] and BirdLife Australia Working List [7].

Strong territoriality is vital to many avian species. In some species, as in Australian magpies (Figure 1, *Gymnorhina tibicen*, sometimes also called *Cracticus tibicen*), territories are held all year round. While year-round territoriality is rare in high latitudes, some species have evolved into stable [1] and even permanent social groups in permanent territories [2], especially in low latitudes (i.e., Southern Hemisphere and equatorial regions), including the Australian magpie (hereafter referred to simply as magpie).

With some exceptions [1], the well-accepted and prolific literature on neighbour/stranger vocal interactions deals largely with species with seasonal (breeding) territories and small song repertoires. This combination seems more common in high latitude species. At least the majority of studies we have concerning vocal defence against neighbours and strangers tends to deal with species that have relatively small territorial song repertoires and seasonal territories. By contrast, Australian magpies (Figure 1) have all-year round permanent territories, cooperative defence [8], and large vocal repertoires. We know relatively little as to whether those specific pre-conditions may require different ecological and vocal adaptations, an area of research still somewhat neglected [9]. The need to hold on to the same territory may have had profound consequences on vocal behaviour (i.e., repertoire, type, and frequency of communication). There is also a great need for more research on how, when, and what is vocally acquired by juvenile territorial birds.

According to Tobias et al. [9], roughly 18% of all songbirds (of over 10,000 species) use duets and choruses in a territorial context. However, not all species share the same sense, extent, and purpose of territoriality. Seasonal battles over territorial ownership may require different strategies and different relationships with neighbours than extended territoriality spanning years. Interestingly, duetting and choruses are most closely associated with all-year round territoriality and strong social bonds [9].

This observation certainly holds true for Australian magpies. Strong social bonds may be expressed in well-rehearsed techniques of effective territorial defence, and this may occur via a subtle, nuanced, well-timed, and highly developed vocal communications system [9,10]. Maintaining a permanent territory may have substantial benefits, such as stability and a safe place to rear young.

Finally, territorial birds have to assess risks posed by strangers and neighbours, for which Getty [11] used the phrase “dear enemies” taken from Fisher’s [12] concept of neighbourhoods, arguing that one of its important functions lay in creating stability despite potentially competing interests between neighbours. Although skirmishes can break out among magpie groups, an uneasy truce can generally be maintained by vocal means alone (such as individual calling, duetting or calling in choruses). Ydenberg et al. [13] preferred to suggest that such an equilibrium between neighbours may be established not so much by a suspicious and thinly veiled agonistic vigilance, but by a degree of familiarity with the strength and weaknesses of neighbours. In many species, including magpies, familiar calls are those of neighbours often with an overlap in song/calls with other neighbours, confirming their neighbour status and proximity [2].

**Figure 2:**
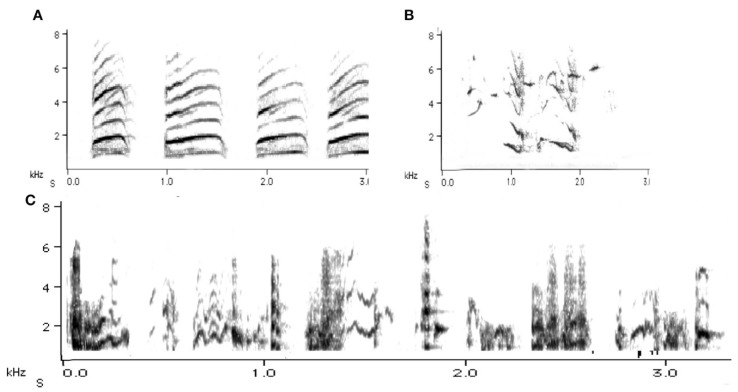
Juvenile vocal expression at fledging. (**A**) Begging call: stereotyped and of high frequency. (**B**) Affective (impulsive), spontaneous sounds (chaotic): expression of fear and distress. (**C**) Well-practised vocal expressions: evidence of the beginnings of a sizeable song repertoire.

Territorial species have evolved a range of perceptual and learning mechanisms that allow them to identify strangers and neighbours immediately [12,13]. One of the classic studies of territorial use of song by Beecher et al. [14] showed that some songbirds, such as song sparrows (*Melospiza melodia*), recognise singers by song type rather than by individual. By contrast, in magpies, territorial references are largely made by calls. Territorial calls are to some extent stereotyped. Their “songs”, usually referred to as warbles (Figure 2), have so far not been regarded as playing a significant role in territorial defence. Thus, it remains to be answered whether and how strangers are actually recognised. These are very old topics and questions in ethology [12,13,15].

However, while we have countless studies on how songbirds learn to perfect song in order to attract a future mate, there is a dearth of literature on how territorial juveniles actually acquire a sense of what is socially appropriate calling, what to listen for and to express within the natal territory. When neighbour and stranger vocalisations are voiced, presumably juveniles have to learn when to act and what signals to use. In juvenile vocal development of territorial species, it raises questions not only about what the birds learn but also when and under which psychosocial conditions they may express, or have to suppress, such vocalisations.

The Australian magpie makes an excellent study subject for such developmental questions in territorial birds. The vocal development of nestlings has been studied in detail [16,17]. We know that juvenile magpies emerge from the nest with a sizeable song repertoire as well as with the ability to produce powerful, high-frequency begging calls (Figure 2). They need this voice specifically in the first few weeks post-fledging because they only tend to fly short distances, land somewhere, then call, and the adults fly in to feed, mostly well away from the ground. Then there is a switch once they are better flyers and juveniles suddenly go to the ground after 12–14 days post-fledging, walking next to the foraging parent [18]. Juveniles usually stay in the natal territory for another 7–9 months post fledging, giving researchers plenty of opportunity to study any development and/or increases in vocal repertoire and in vocal and social reactivity to the importance of territory.

For adults, we also have substantial information about their vocalisations and know that magpies, unlike most seasonally territorial songbirds so far studied, have large repertoires. Repertoire size can vary within a species and it seems that those territorial birds with larger repertoires also have more reproductive success, as was shown in song sparrows, *Melospiza melodia* [19]. In a comparative sense, “large and small” are rather relative terms, however, and they may be measured in different ways. Indeed, what constitutes a “large” repertoire is not as easy to define as it appears. A species may develop a set of different songs whose structure and content may be fixed in each case (stereotyped song). Repertoire size is then measured in the number of songs the bird has, as is the case in song sparrows or the nightingale, for instance [20]. Or repertoire size may be determined by counting discrete sequential clusters (phrases that remain fixed and together but may be moved to different parts of song). Finally, and this is relevant to the magpie’s repertoire, one can measure repertoire size by the number of syllables used.

**Figure 3:**
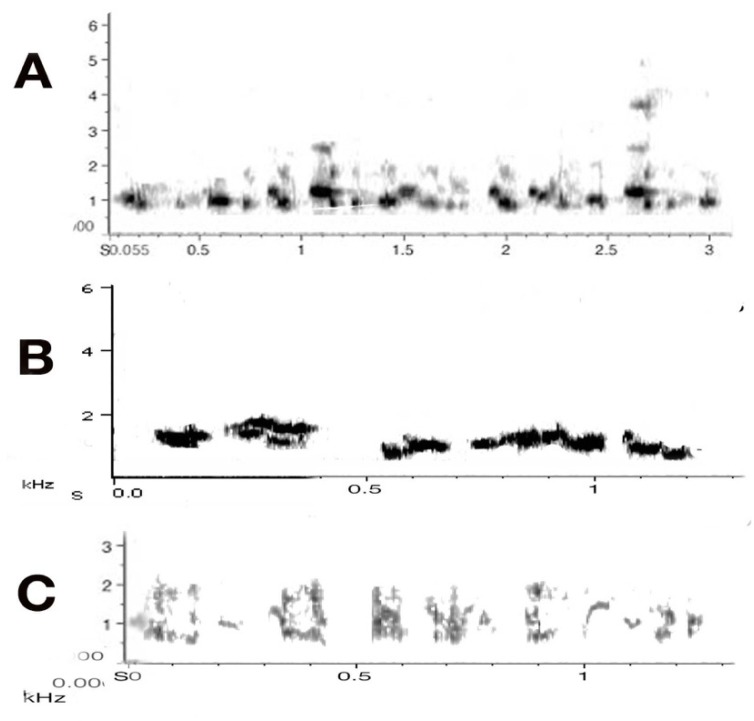
Adult and juvenile warble songs. Y-axis: frequency in kHz, X-axis: time in seconds. (**A**) Upper-panel: typical song/warble sequence by an adult. (**B**) An enlarged version of the same (1 s excerpt). (**C**) Enlarged 1 s of a juvenile warble, still revealing recognizable structures in elements and syllables. Warbles are produced in a narrow frequency band, as (**B**) shows, and have relatively few or any overtones (harmonics). Such songs can be performed for hours [2,23]. The magpie’s song repertoire has been estimated to contain up to 893 syllables [24], and, at times, includes mimicry [25].

Syllable repertoire perhaps best describes the magpie’s song. Syllable repertoire has no discrete sequential clusters and is most common in continuous singers [21]. Some 40 years ago, Catchpole [22] commented on the song of sedge warblers, *Acrocephalus schoenobaenus*, that they may never sing a single song twice in their lives because each song is long and the elements follow each other in highly varied order. The magpie’s song is continuous, and the elements may be arranged in different ways each time. Magpies may thus be described as continuous singers. Indeed, some of their song bouts also can be extremely long. Their songs are soft and undulating sounds (Figure 3) and because of these qualities, the magpie’s song has usually not been considered as having a role in territorial defence.

**Figure 4:**
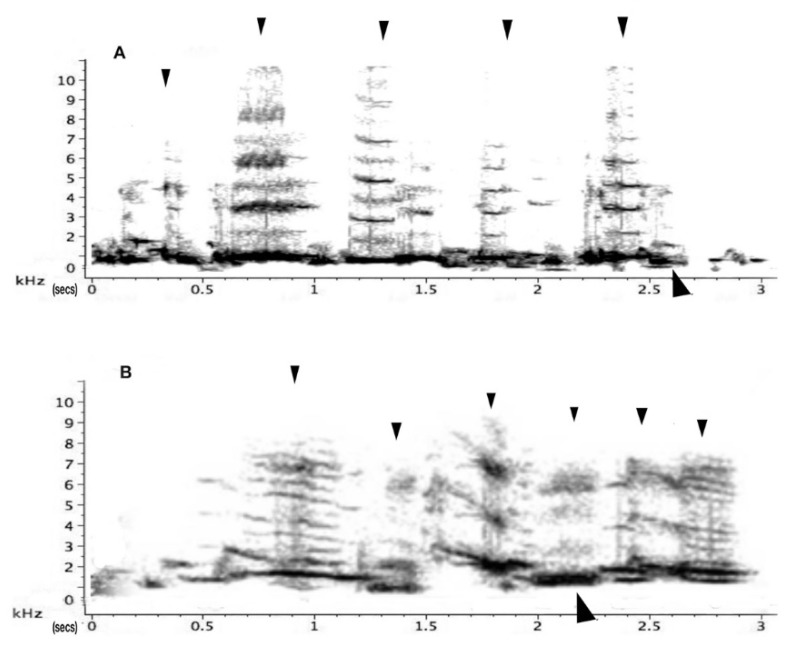
Two examples of a carolling bouts (from birds from different territories). Y-axis: frequency in kHz; X-axis: time in secs. Black arrows facing downwards indicate a single sound; arrows from below identify the first formant (dark area). Formants are frequency peaks in the spectrum which have a high degree of energy. In the first sample (**A**) harmonics are well defined, giving the carolling a tonal quality. In (**B**), however, the sonogram is fuzzy and grey, indicating that the sounds produced are noisy and harsh. In most songbirds, upper harmonics (parallel lines above first formant) usually have little energy. In carolling, however, there is still substantial energy (i.e., are audible at 6 kHz (**A**) and even 7 kHz (**B**)). The darkness of the trace is proportional to the energy (or amplitude) of the signal. Sound amplitude is experienced as the loudness of sound. The ability of magpies to vary sound intensity depends on vocal tract manipulation and even on its length [30].

In adults, the very melodious songs (warbles) as presented in Figure 3 are in stark contrast to the main territorial call, the carol (Figure 4). Earlier experiments by Carrick [26] had already confirmed that this this particular call type, called carolling (Figure 4), is used specifically to advertise and defend territory. Carrick removed the male from the territory and instead played pre-recorded carolling calls of this particular male. Despite being surrounded by neighbours on all sides, daily playbacks of a male magpie’s carolling calls alone over several consecutive weeks maintained the territory [26,27]. Some ten years later, the first speaker replacement study undertaken in Europe (Sweden) found that in thrush nightingales, *Luscinia luscinia*, territories were held for as long as 10 days on the basis of playbacks of territorial songs alone. However, this was for a period of 10 days at the most [28]. Eventually, these territories were invaded. When Krebs [29] repeated such playback experiments in great tits, *Parus major*, a few years later, he found that playback of territorial songs alone could hold the territory but for a relatively short period of time. Catchpole and Slater [20] suggested that the vocal bluff eventually had to be followed by some visual proof of the existence of the male, and if this was not forthcoming then more daring males took over the territory. Magpie carol playbacks held the territory longer than playbacks in the above-mentioned European songbird studies. The different results between the playback experiment with magpies and those using thrush nightingales and great tits may be one of territory size. A single magpie pair may occupy several hectares (even up to 100 hectares/1 km^2^) in inland Australia) and may well be and stay out of visual range for a good while longer than woodland birds may be able to do in relatively small breeding territories. Territory size is presumably one reason why carols need to be so loud (Figure 4).

**Figure 5:**
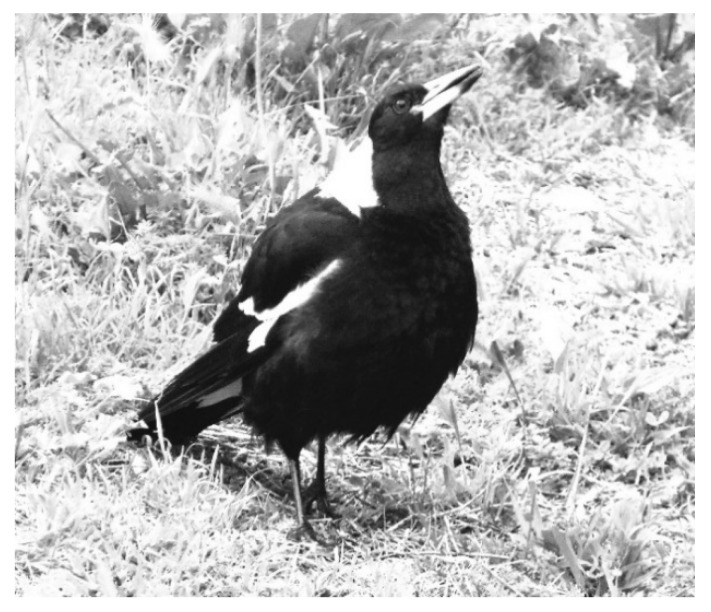
Male beginning to carol: typical posture seconds before the onset of carolling. Note the raised and ruffled looking feathers particularly on the upper and lower breast and belly indicating muscle involvement in a call that may take a good deal of energy, and a second before, the head is thrown back and the beak opened wide.

Indeed, the functional and biological aspects of territorial calls in magpies are now well documented [16,31,32]. Carolling bouts are used by males and females alike and pairs often duet, prompted either by the male or the female [33]. A casual and usually very brief carolling event may erupt amidst other activities such as foraging [3], and they may be seen perched on a branch on their own but more frequently they carol when on the ground (Figure 5).

**Figure 6:**
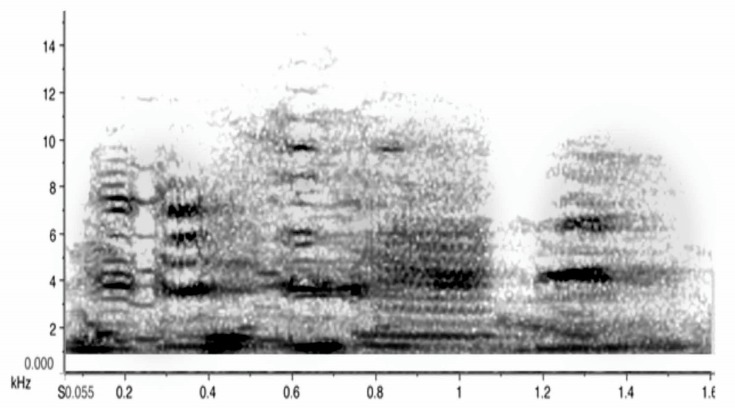
A 1.6 sec excerpt of a group chorus by three magpies in overlapping carolling bouts. Note that the sound is almost continuous and very noisy (fuzzy grey) but it still has strong energy in the 4–6 kHz range. It is of high intensity measured at 90–100 dB at 10 m distance, (similar in intensity to the sound of a jackhammer or a fast-moving freight train). The loudest territorial duets and choruses in land animals are usually associated with primates. In howler monkeys, for instance, these have been measured at around 6–7 kHZ and 88 dB at a 5-m distance [37], with similar readings of duets or choruses in other primates [38,39,40]. In magpies, I have measured magpie group choruses at 90 dB and even 100 dB at a distance of 10 m.

However, magpies also come together to perform group choruses (when the group consists of several adults) [33]. In such instances, the adults fly to an exposed position in a tree, on a roof top, overhead wires, TV antennas (see Figure 12). As in kookaburras [34], such family group gatherings occur after the successful eviction of an intruder or a bird of prey. A group chorus of magpies may well be amongst the loudest of any land animals (Figure 6). This chorus or group carolling is thought to be a pronouncement of victory, possibly meant to strengthen the group’s bond, as reported in other species [35] or at least, as in kookaburras, maintain the social hierarchy [36].

The importance of such choruses in territorial songbirds, as Tobias et al. [9] rightly argued, may have been underestimated and not investigated enough, as mentioned above.

At least carolling has been closely studied and is known to have a key role in territorial defence [26,27]. It is also a very distinctive call and is produced by adult magpies adopting a visibly distinctive physical posture (as Figure 5 shows). It would seem reasonable to assume that this call and possibly others associated with territoriality would be particularly important for juveniles to acquire and for them to learn when it is appropriate to apply specific vocal signals. Moreover, there tend to be additional pressures on the signal characteristics in territorial calls. As Nelson [41] in his study of white crowned sparrows, *Zonotrichia leucophrys pugetensis*, recently pointed out, complex signals that convey diverse forms of information may face conflicting pressures on their structure. He argued that certain messages, such as species identification or “alerting” receivers, may, on the one hand, “require a relatively invariant signal structure, while messages about individual identity and motivation on the other require structural diversity within and among individuals of a species.” [41].

While Nelson’s observation is pertinent here, the process by which invariant and flexible signal structures are acquired remains almost unknown in many species. The point of this study was thus to identify clearly when and what juvenile magpies learn while in their natal territory and whether one can establish whether they acquire a sensitivity to more complex social signals and can assess their relative importance in maintaining a territory.

The project reported here falls into three parts: Phase 1: collection of all adult vocalisations (as far as possible) from all focal territories and identifying calls by function. Much of this work was done before the project commenced and the two-year sampling served only to confirm the type and number of calls already known and catalogued. Phase 2, the bulk of this project, consisted of collecting calls and songs from juveniles over the research period (one breeding season) in order to compare their vocal output and types of calls used with that of adults. Phase 3, consisted of a field experiment with playbacks of calls of neighbours and strangers inside their respective territories, testing individual adults and juveniles in their responses to these calls. These neighbour/stranger playbacks were used to test both adults and juveniles separately. Test periods for juveniles started early post fledging (week two) to week 14 post fledging, providing an opportunity to observe and score developmental changes in their responses.

## 2. Materials and Methods

### 2.1. Subjects, Time Frame, and Location

For Phase 1, the first study was conducted over a three-year period using 20 nest sites recording the number of times a nestling vocalised in relation to the absence or presence of any adult or parent bird. Vocalisations were subdivided and scored separately for begging responses and for song practice. This first stage was not included in the present paper because it has been fully reported elsewhere [17], but knowledge gained from this study acted as a baseline to proceed to the core interests of this study.

Of the 20 different territories, five sites were chosen for further focal study. The territories were selected in accordance with several basic principles: a) accessibility, b) known borders, and c) distance relative to each other (excluding neighbouring territories on three sides) and preferably only two or three offspring per territory (no helpers from a previous year) in order to be able to observe and record all of their vocal behaviour. All research was conducted in the field, at (30°32′S, 151°40′E). latitude and at an altitude of 800–1100 m, on the Northern Tableland in New South Wales near the university town of Armidale with very suitable habitat for the species, such as open woodlands with some mature trees and access to water sources.

### 2.2. Recording Vocalisations of Adults

All sound types (i.e., calls, mimicry, song) used by adults had already been collected over a ten-year period providing a substantial library of magpie vocalisations. A major advantage in studying magpies in the field is that, unless some mishap occurs, the same magpies stay in the same territory. Hence, the acoustic characteristics provided here (there are variations within each group as well) were derived from the same magpies and the same five magpie territories in which the study of juvenile vocal behaviour was conducted. Hence, recordings and sonograms of their substantial repertoire of adult calls and song were already available at the time of commencing the study of juvenile vocal development with special emphasis on territorial calls. These data form the background on which comparisons between adult and juvenile behaviour have been made possible.

However, during the project on juvenile vocal development and communication, sound recordings of adult vocalisations were repeated in case the same magpies had added or changed any aspects of their vocal behaviour. Over the 14-week period, all acoustic profiles of the five focal magpie groups were identified and catalogued. Note that the detailed acoustic elements were exclusively derived from the adult members of the group. Songs and calls from focal adult male and adult female magpies in the five territories (*N* = 10) were catalogued over two breeding seasons, adding the vocalisations in the second year just in case of additions or changes in adult repertoire. Thus, a substantial set of adult vocalisations was already available when juvenile vocalisations were recorded and itemised. Each territory consisted of one adult pair only plus offspring.

**Figure 7:**
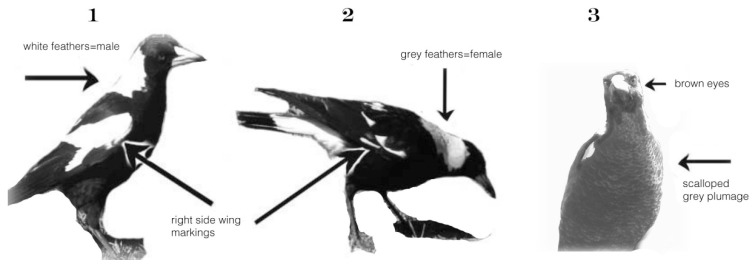
Identifying magpies in the field is generally simple. Wing-markings are obvious, individually distinctive, and unchanging over the lifespan (see lower arrows in 1 and 2). (**1**) Shows an adult male, identifiable by the clear white feathers at the back of the neck; (**2**) is an adult female, the only difference in plumage is a small strip of grey (as per arrow) at the nape of the neck; and (**3**) is a juvenile with greyish scalloped plumage. Adult plumage overall is black, interspersed with white. Note that wing markings in adults and also in juveniles (no separate juvenile stage for tail and wing feathers) are individually specific. Juveniles are distinguished by darkish brown eyes (adults have light brown to reddish eyes). Juveniles cannot be sexed by plumage.

Individual adults did not require tagging because their white wing-markings are obvious, individually distinctly different and remain the same over the lifespan. The sex of black-backed magpies is identifiable by the feather colour at the nape of the neck (Figure 7). In males, it is bright white, in females, there is a small grey strip of feathers between the black and the white. Juveniles cannot be sexed before the first moult, but they are easily distinguishable as juveniles by their grey, and on the chest, scalloped plumage (see Figure 7 [3]).

Observations and recordings were made in a rotational 2-h observation period per group per day (between 6.00 h and 16.00 h daily, five days a week). All call types observed and recorded were transferred from a Sony Field Cassette Recorder (Sony TC-D5 PRO II, Tokyo, Japan) to computer. Using Raven Pro software (v.1.4, The Cornell Lab of Ornithology-Bioacoustic Research Program, Cornell University, Ithaka, NY, USA) they were transformed into sonograms.

Vocalisations were categorised first purely according to acoustic characteristics of individual singers, not according to function. It was merely noted in which context each acoustic signal occurred. This made it possible to objectively compare sound elements and syllables between adults and juveniles.

### 2.3. Recording Vocalisations of Juveniles

The question that has tended to be entirely neglected is what and when juveniles learn about territoriality as a concept, meaning when and how they learn/are able to distinguish between neighbour and stranger calls and songs, and whether they begin to attribute any importance to such signals.

Juvenile vocal expressions were recorded over the entire period from the day (or near) the day of fledging up to and including 14 weeks post-fledging, between early September and mid-December (during the second season of recording adult vocalisations). After 12 weeks, post fledglings are able to find most of their food by themselves, while they are usually competent and entirely independent feeders by week 14.

**Figure 8:**
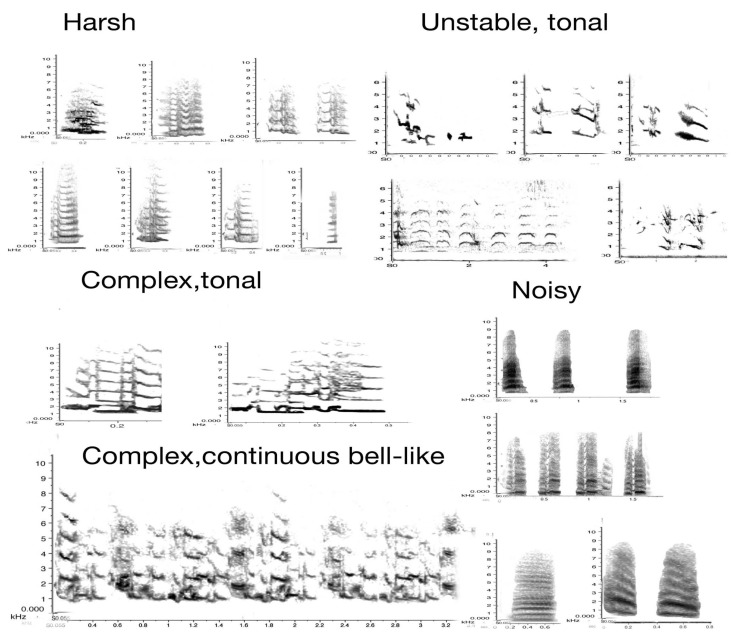
Acoustic profile of adult magpie vocalisations. Y-axis: frequency standardised to 10 kHz, except for the group of unstable sounds (standardized at 6 kHz). X-axis: time in seconds. Individual calls or sounds of 0.2–0.5 s. The exception in this figure is the “complex/continuous” sound that was recorded over 32 s.

Vocal expressions by juveniles were recorded by date and type for each individual and the results also rendered as sonograms to match adult sound profile where possible. These emerging vocal developmental data are summarized in Figure 8 and Table 1 below (for details of the time-frame of their expression see Reference [17]; for the physical abilities in such song production see References [30,31].

### 2.4. Playback Experiments

As the second part of the field work, responses to vocalisations were tested experimentally for both adults and juveniles. Playback sequences associated with territoriality were prepared in order to score the responses of adults (*N* = 10) and those of juveniles (*N* = 12) to these playbacks in the same five permanent territories in which all vocalisations had been initially recorded.

#### 2.4.1. Playback Preparation and Details

The playback preparation included three separate and carefully devised sequences consisting either of song alone, of song interspersed with carolling bouts, and of carolling only, either as a singular call or as a duet. These were prepared separately for neighbours and for strangers. The samples had been recorded in the field. Each playback emphasised a different category of vocalisations produced by the same neighbour or stranger (i.e., as said above, consisting of warbles or of warble/carols or of carols). The playback recordings purposely differed for each test group in that neighbour calls were carefully chosen to be the closest to each focal group and not a shared neighbour with any other group. Thus, five neighbour playbacks were prepared but only three stranger vocalisations were recorded (far enough away from any of the five focal groups to have remained unknown vocally) and rotated around the groups in the three trials, offering, in fact, a “new” stranger set of sounds in each trial. In all five groups, adults had thus listened to any specific stranger playback just once (avoiding habituation).

The samples were cleaned and standardised into 5-min playbacks with breaks between calls no longer than 5 s. Cleaning the samples involved removing any background noise (e.g., cars, people, dogs) and any unclear song; the calls or song segments themselves were left untouched so as to preserve the characteristics of the individual’s song or calls.

The segments selected were actual segments of neighbouring or strange birds. Hence playbacks were as characteristic as possible of an individual bird from another territory (either a neighbour of the test bird or a stranger) and each playback consisted of at least one repeat of the recorded bird’s most typical phrase.

For each playback preparation and for each type of call, the decibel (dB) output was also measured (at a 10-m distance from an adult bird). This measure was used as a guide for reproducing the same sound intensity in dB. We used a decibel meter, specially designed for measuring outdoor/environmental sounds and noises to achieve the right amplitude at 10m distance and account for attenuation of sound through air. Since Marten and Marler’s [42] excellent study of sound transmission for field studies in animal vocalisations, we know that excess attenuation is greater at ground level than at other heights, and more so in open habitats than wooded ones, and that the highest loss of intensity tends to occur with the highest frequencies. All three variables applied in this project and had to be taken into consideration: the playback unit was placed on the ground, territories were open woodland, and many of the calls were high frequencies.

Thus, each focal magpie was exposed to playbacks from a stranger and a neighbouring magpie. A trial consisted of a 5-min silent pre-test, a 5-min playback (either a stranger or neighbour), and a 5-min post-test silence. Vocal behaviour was scored for each magpie during this 15-min period. Specific responses by adults were also recorded by pen and paper such as flying, duetting, and vigilance behaviour.

Playback was delivered from a small digital Zoom H2 Handy Recorder, a digital field unit (only 10-cm high, without stand) (see Figure 10), capable of strong output, and thus not requiring an external speaker. Decibel range was at 65–75 dB, mimicking the frequency range of warbles and warble carols (varying within a single playback) and at 85 dB for carolling both at a 10-m distance.

Scoring juvenile responses also included a range of non-vocal responses such as changes in visual direction (i.e., looking up, down, around), altered locomotion patterns (i.e., flight, walking, running), changes in body posture (e.g., crouching, stopping foraging, standing erect and other typical vigilance postures), and reference to adults (i.e., looking at or running towards parents).

#### 2.4.2. Number and Time of Sessions in the 3 Trials

Three playback trials were run per individual, approximately one trial per five weeks between spring (September) and early summer (December). A trial consisted of two playbacks to one focal individual per test day, separated by at least five hours: one playback of neighbour calls/song and another of a stranger. Three trials were necessary because the playbacks were different on each occasion: consisting of carols only, or of warbles or of warble–carols only.

The playback experiments thus resulted in a total of 132 sessions presented over a period of nearly four months: involving three trials, using a total of 22 individuals with two sessions each (neighbour/stranger), yielding 44 sessions × 3 trials, i.e., a total of 132 data points. The sessions per trial had to be randomised between the five territories and the kind of playback to be used (warble/carol/carol–warble).

#### 2.4.3. Conditions of Playback

In these field experiments, it was important to set some strict conditions. These included placing the playback unit 5-m inside the borders of the subject’s territory for playbacks with juveniles and adults. A trial only commenced when a focal magpie was at a visual distance of about 10 m and was not engaged in any particular activity (such as fighting, flying or foraging). Only then the experimenter started the playback unit and did so remotely. If these conditions were not met on the stipulated day, one needed to come back to the site as close to the time-tabled time as possible; hence, the 22 days often turned into 30 days but the additional “free” eight days were built into the design to follow up on individuals. Experiments that were interrupted by another magpie flying in or beginning to engage the focal bird had to be terminated and were not counted. If other magpies were visible but did not engage with focal magpie, the test was conducted, but any vocal behaviour following the presentation was only scored with the focal magpie. In all, presentations in any specific location occurred only once every four to five weeks. This was meant to avoid habituation although running vocal playbacks in the bird’s natural environment unavoidably meant that even though territories were large, no doubt sounds were overheard by others in the group even without visual contact, and in the case of playbacks for juveniles, at least one adult bird tended to be in visual distance of a juvenile.

All procedures used were approved by the University of New England Animal Ethics Committee (AEC08/81) and were in accordance with the Australian Code of Practice for the Care and Use of Animals for Scientific Purposes, edition 7 (National Health and Medical Research Council, 2004).

## 3. Results

### 3.1. Acoustic Profiles of Adult Vocalisations

The acoustic elements presented here are exclusively derived from the adult members of the five focal groups. The main aim of this part of the project was to identify all adult vocalisations and their sound structure. The main vocal categories out of all the acoustic characteristics collected above, as already discussed, are carolling and song, both very well researched, [2,16,23]. Carolling, the main territorial call, is a short but powerful slur. It rarely exceeds 3 s in duration but may be repeated in duets by alternating between birds, and when more than two birds are involved (chorus), may exceed 100 dB in amplitude (see Figure 4 and Figure 6 above).

The sound profile is presented in sonograms in Figure 8 below. It includes all vocal elements collected and uttered by any adult, with two exceptions: the song/warble and the main territorial calls have already been introduced and are thus not included in Figure 8. Note that these are organised by *acoustic* characteristics rather than by function. The sonograms also indicate that great and subtle variations in sounds can be achieved by increasing noise or amplitude alone.

**Figure 9:**
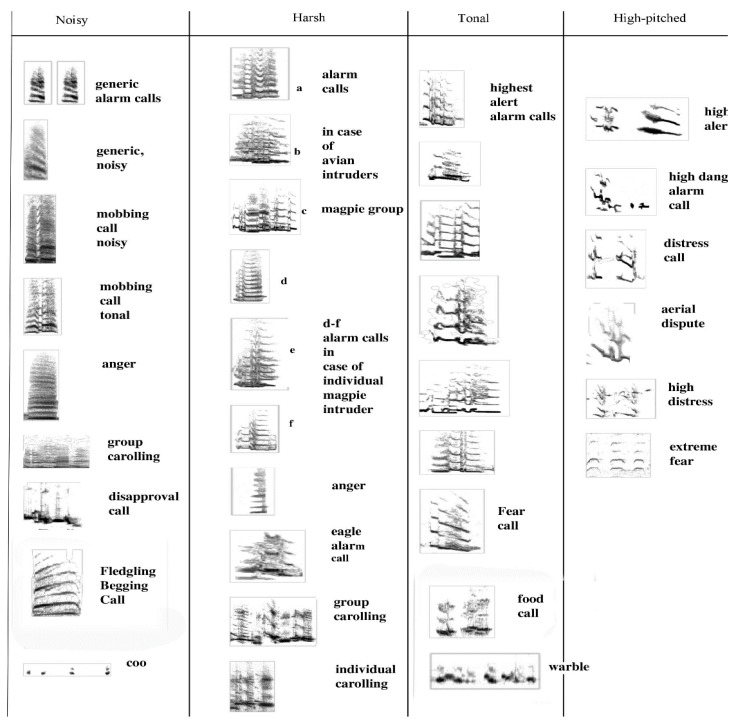
Acoustic characteristics of magpie vocalisations: 33 distinct sounds that, even to the human ear, have distinct sound profiles. The context and function are identified next to the call. **Column 1**: (noisy calls), note the degree of noise (grey) in each call and the high amplitude. All these calls are meant to attract attention. Mobbing calls are rallying calls to get all group members to attend to an intruder. The begging call of nestlings is already of an amplitude similar to that of adult mobbing and alarm calls, first formant about 1.5 kHz but with main energy still at 3 kHz. The low amplitude “coo” call is specific to a female entering the nest with live young. It is possibly the lowest amplitude sound produced by a magpie ever recorded, and was only audible at all because a microphone had been attached to a tree, next to the nest, some 30 cm away from the nestlings (measuring just 25 dB). **Column 2**: shows a number of warning calls (a–c predatory intruder; d–f neighbouring/stranger magpie intruder), including the eagle alarm call [45]. The last two calls are territorial calls, issued in pairs, as a group or individually. **Column 3**: contains complex, very melodious well-defined, and even near flute like sounds, largely devoid of noise. They may consist of initial arousal calls, refer to expressions of fear, or even be referent (such as the call suspected to be a food call). **Column 4**: contains very high-pitched snippets of sound of affect (i.e., fear and distress). Extreme distress calls were identified when a bird was about to be killed by a bird of prey (the observer intervened, and it did not happen).

These samples are presented with frequency and time span to indicate their defining characteristics. In the next figure (Figure 9), only the images of the same sonograms are presented. The importance of Figure 9 lies in its link to function. While functions can often only be inferred judging by the behaviour that consistently preceded or followed a specific call, some of these have been subject to extensive field tests. Some alarm calls have been studied in detail [43,44,45]. For instance, the referential signal we termed “eagle alarm call” has been experimentally confirmed [45,46] and discussed as a referential call [47]. Linking calls to function is vital in trying to interpret what juveniles can and will do vocally over time within their natal territory.

Figure 9 has grouped the same sounds by function, not haphazardly, but based on extensive recordings and observations over a decade. In combination with other calls, the use of such calls may well change. We are just at the beginning of understanding how complex magpie communication can be. For instance, one of the tonal calls, identified in the third column as a food call (i.e., potentially another referential call), occurred only in contexts of provisioning an individual magpie. The call sounds like a somewhat mellow territorial call but once recorded and transferred to sonograms, it became clear that it was entirely different in structure and that, upon receiving a morsel of food, the calling would bring other magpies to the site of the provisioning.

### 3.2. Acquisition and Use of Repertoire by Juveniles

In the five territories, there were a total of 12 juveniles (three territories had produced three offspring each, one territory had two offspring, and the fifth had one offspring). Repertoire acquisition in juveniles was relatively difficult to ascertain in some cases because some sounds were rarely heard or expressed. This part of the project started with the premise that juveniles (post-fledging) were vocally competent. This was not an assumption but based on detailed previous studies that had identified the acoustic elements practised in nestlings and/or heard in the first few months post fledging [17].

**Figure 10:**
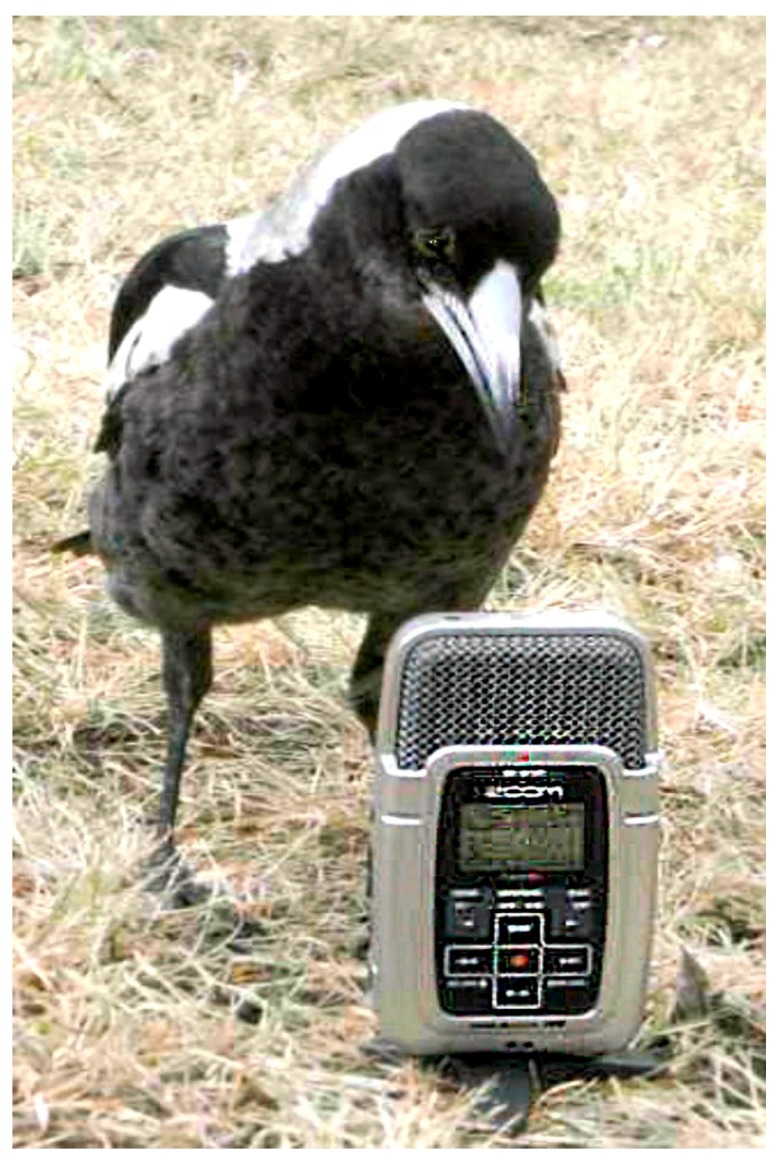
Juvenile magpie examining the small digital playback unit positioned on the ground [48]. Note that the speaker (top grid) is two-sided and works on either side of the unit. Here, the juvenile’s inspection occurs on the side from which the sound emanated.

Juveniles are also very curious, and a number of playbacks were unsuccessful (no score) in the sense that the magpie concerned, unlike the adults, showed more interest in the source of the sound rather than the potential nature and status of the playback material (Figure 10).

Results showed that juveniles, within the first three months post-fledging, expressed vocalisations in the same frequency and amplitude range in which those of adults typically were, but did not develop the entire range of calls and acoustic profile of adults. Using sounds that were within the same range of amplitude and frequency as those of adults indicates that there were no obvious physiological and maturational constraints for juveniles to use the entire range of adult vocalisations. However, they did not.

In terms of actual production of sounds, there was a large gap between those voiced by juveniles and the sum total voiced by adults. Table 1 below lists the sounds that were actually produced by and recorded from juveniles. This shows that juveniles produced only a fraction of the calls of the adults, even by the fourth month post fledging.

Only one category was clearly maturational, namely the begging calls that first developed in nestlings and had reached their auditory peak and clear structure by the fourth week as nestlings. Begging calls decreased over the study period as juveniles became more competent in finding their own food. Another might have been an alarm call. The voicing of a general alarm call, derivative of the generic alarm call but not of the same amplitude, increased over that period. These calls were largely directed at other juveniles. They concerned minor daily events, nearer to annoyance calls, i.e., expressed when one juvenile snatched some food away from another. This type of call was also voiced when spotting an object that might have been harmless but novel for the bird. Such alarm calling tended to be shorter than the generic alarm calls by adults, had almost the same structure as the generic adult alarm calls [3].

These results were a surprise. They showed that the majority of calls used by adults were never uttered by juveniles at any stage of development and some other calls were rare or only very occasionally used by juveniles (see Table 2). One specific call, carolling, was produced just once by two juveniles, each belonging to a different group. In the other three groups, carolling calls by juveniles were not recorded, and in those two groups in which a juvenile was vocally expressing a carolling call, adults responded immediately, intercepting and punishing such behaviour.

In summary, the results on vocal behaviour collected from 12 individual juveniles from five different territories, showed that juveniles produced only 11 of the 26 calls. Given the high number of unvoiced sounds by juveniles (note the large number of the N (Never) category in Table 1), it is of particular importance to examine these sounds in terms of their function.

As was shown in Table 1, juveniles did not participate in noisy, harsh or highest alert alarm calling, noisy or tonal mobbing calls, in group or individual carolling. They did not issue high alert or high danger calls and never uttered coo, or high danger or food calls, and never expressed disapproval.

Thus, the only sounds expressed by juveniles were those concerned with personal well-being and emotions (i.e., anger, fear, surprise, annoyance) usually in the context of the company of other juveniles. Importantly, not a single syllable of the juveniles’ repertoire related to territoriality or danger.

There were two exceptions. Two juveniles from different territories were each once observed trying to carol, i.e., using the territorial call. Both were also swiftly punished by the adults. Such a rare behaviour is important to remember in so far as it might shift one’s interpretation about the absence of entire vocal categories in juvenile calling. The fact is that juveniles did not voice calls that were directly or indirectly connected to territoriality and danger. The two exceptions of carolling attempts by two different juveniles were at once followed by rebuke from adults.

### 3.3. Playback of Neighbour/Stranger Call

Given the absence of any juvenile vocalisation concerned with territory, the playback experiments became more important than had been initially appreciated.

#### 3.3.1. Response to Playbacks by Adults

Playbacks to adults (*N* = 10) elicited definitive, distinct, and prompt vocal responses, and more than that, each type of playback elicited important and statistically significant results in each category (i.e., warble, warble–carol, and carol). All behavioural data for each time period were checked for normality. Only scores for warble behaviour needed to be square-root transformed which achieved a normal distribution. Analysis of variance (ANOVA) tests were applied to the behaviours scored, with testing period as a repeated measure and playback type a factor. *T*-tests (2-tailed) were used in the post hoc analysis, the non-parametric equivalent of the independent t-test (Mann–Whitney U) and paired t-test (Wilcoxon) used on data that had multiple zero scores were analysed by non-parametric tests, which was necessary for carols only.

**Figure 11:**
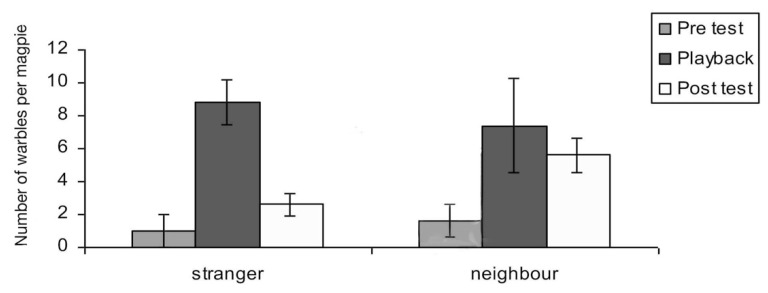
Warble responses by adults (*N* = 10) to playback of warbles by neighbours or strangers. Graph indicates mean number and standard error of all adult warbles in response to warble playbacks. Note the importance of the post-playback period, showing that the alert vocal response continued after hearing a neighbour sing inside their territory, and there is a significant difference (*p* = 0.002) between responses in the post-test period between hearing a stranger or a neighbour warble.

Warbles: “Songs” or “warble” were identifiable as soft and often sounded of relatively low frequency (2 kHz), as was shown in Figure 3 above. There was a significant main effect of test period on warbles (F_2,10_ = 9.04; *p* = 0.002). Warbles increased during playback of both neighbour and stranger vocalisations. (pre-test to playback-period, *t* = −5.087, *p* = 0.007). There was also a significant interaction between period and playback of neighbour versus stranger vocalisations (F_2,10_ = 3.85; *p* = 0.043). In the post-test period, warbling was significantly higher for playback of the neighbour’s vocalisations than for playback of the stranger’s vocalisations (*t* = 0.710; *p* = 0.037). Warbles in the post-test period of the tests with playback of neighbour’s calls were also significantly elevated compared to the pre-test period (*t* = 5.464, *p* = 0.005). For neighbours’ warbles, the main effect was a continuation of warbling well into the post-test period (Figure 11).

**Figure 12:**
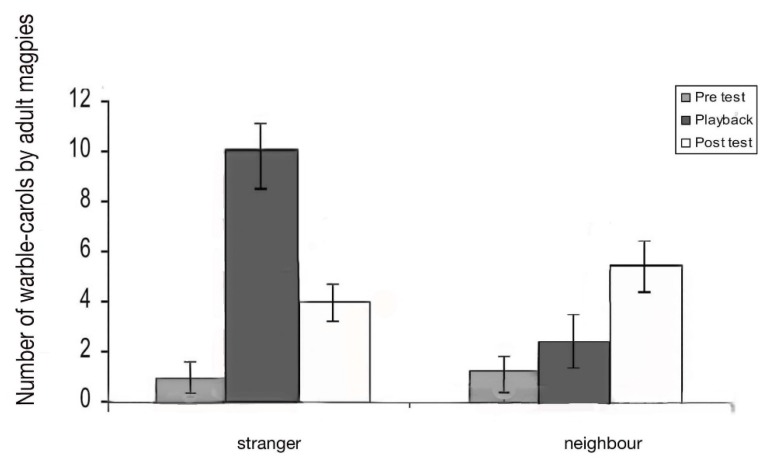
Vocal response by adult magpies (*N* = 10) to warble/carol playback over a 15-min test period. Response to playback of the call of a stranger is significant. Vocal responses during playback dropped off quickly once the sound stimulus was removed (*p* = 0.03). To neighbours there was barely a response during playback but a stronger response after the stimulus removal (*p* = 0.018).

Warble–carols: Warble–carols are a distinct form of vocalising that mixes song (“warbles”) with interspersed carolling calls (Figure 9). There was a significant main effect of the test period on warble–carols (F_2,10_ = 14.94; *p* = 0.001). There was a significant interaction between test period and stranger and neighbour playbacks (F_2,10_ = 12.25; *p* = 0.001). Playback of the stranger’s vocalisations led to an increased number of warble-carols during the playback period compared to pre-test (*t* = −3.066; *p* = 0.013) and then a decrease in the post-test period (*t* = −3.639; *p* = 0.005). (Figure 12). Playback of the neighbour’s vocalisations did not affect the frequency of warble-carolling while the playback was heard by the bird, but it did lead to an increase in these calls during the post-test period (comparison of post-test to playback, *t* = −3.882, *p* = 0.018; comparison of pre-test to post-test, *t* = −3.994, *p* = 0.017).

In other words, the birds emitted warble-carols when they heard a stranger. Playback of the neighbour’s vocalisations had little effect during the playback period, but there was an increase in warble-carolling in the post-test period (comparison of playback test to post-test, *t* = −3.882; *p* = 0.018; comparison of pre-test to post-test, *t* = −3.944; *p* = 0.017). In other words, the test birds emitted warble–carols when they heard a stranger. In contrast, when they heard a neighbour, they used warble–carols at any increased rate but specifically in the post-test. During the playback period, the birds produced significantly more warble–carols in response to the stranger than to the neighbour (paired *t*-test, *p* = 0.045). There was no significant difference in the number of these calls produced to neighbour versus stranger in the post-test period (paired *t*-test, *p* = 0.565).

**Figure 13:**
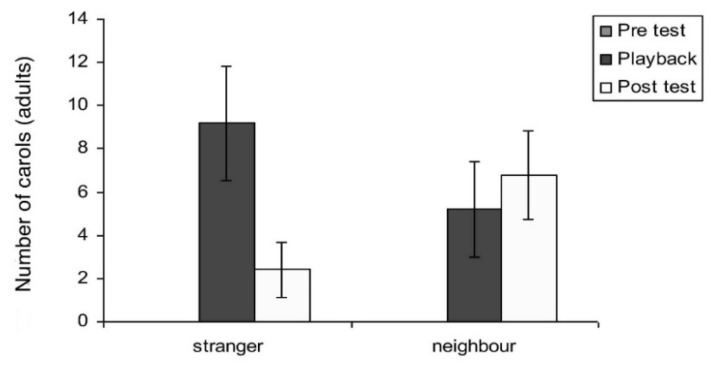
Vocal response by adult magpies (*N* = 10) to carol playback over a 15-min test period. As in warble–carols, the post-test response was significant when comparing responses to carolling of strangers and neighbours. The mean of three trials played inside the territory of focal magpies (for the post-test period: F_2,10_ = 10.43; *p* = 0.001). Graph indicates mean number and standard error of all adult warbles in response to carolling playbacks. Note again the importance of the post-playback period, showing that the vocal response continued after hearing a neighbour carol inside their territory (no significant difference to test period, while the incidence of carolling to a stranger drops off quickly, i.e., in the first minute of the post-test period. Well before the post-test period ended any carolling to a stranger had ceased while, in the 5 min of the post test, carolling continued throughout in response to a neighbour.

Carols: Carols, as has already been described (lower panel of Figure 1), are purely voiced to assert territorial claims. There was a significant main effect of test period on carols (F_2,16_ = 10.43; *p* = 0.001). A not quite significant trend for the interaction between test period and playback type was seen in carols (F_2,16_ = 3.46; *p* = 0.056). However, for carolling behaviour, Mann–Whitney independent and Wilcoxon tests were applied since the data were nonparametric (no carolling was recorded in any of the pre-test periods). These tests showed an interaction between neighbour and stranger playback samples for the post-test period (F_2,10_ = 10.43; *p* = 0.001). In the tests with playback of stranger vocalisations, there was a significant increase in the number of carols from the pre-test period to playback (z = −2.023; *p* = 0.0415). For the tests with playback of neighbours’ vocalisations, there was a significant increase from pre-test to playback (z = −1.826; *p* = 0.034) and pre-test compared to post-test periods (z = −2.023; *p* = 0.0215) (Figure 13). Thus, on hearing the neighbour playback, the birds carolled and continued to carol in the post-test period. However, comparison of response to stranger’s versus neighbour’s calls showed no significant difference in the playback period (*p* = 0.310) or during the post-test period (*p* = 0.169).

Interestingly, adults responded significantly more spontaneously and promptly when exposed to stranger vocalisations. However, the strong vocal response also diminished rather quickly at the end of the playback (see Figure 13). In the playback of a neighbour’s calling, however, responses of adults remained elevated in the post-test period. Adults continued to carol and even more so in the post-test period. By carolling over a ten-minute period, the territorial adult certainly issued a more prolonged reminder that the territory was theirs (Figure 13).

An earlier study had identified seasonal cycles for certain vocalisations. This has been tested for warbles/song and for carolling [47]. Warbling/song was notably most frequent in summer and autumn and then dropped sharply with the onset of the breeding season (there are slight differences depending on latitude and altitude) when the previous brood had usually dispersed, By July, when the female incubated eggs, song had declined to its lowest level in the year. The breeding pair usually fell entirely silent. The trajectory of territorial calls is almost the opposite, reaching the highest incidence of carolling just before and during the breeding season and reaching its lowest levels throughout spring and summer [49].

In other words, the soundscape to which juveniles were exposed from nestling to late juvenile stages was sparse in song but rich in territorial calls, giving juveniles plenty of opportunity, and all the more opportunity to learn carolling.

#### 3.3.2. Response by Juveniles to Playbacks

**Figure 14:**
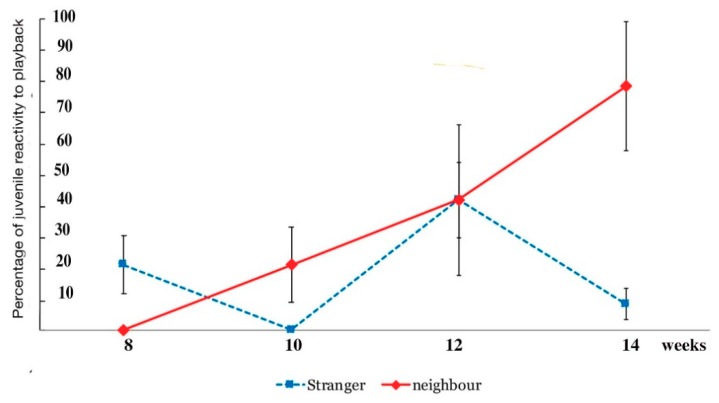
Juveniles responding to playback experiments from weeks 8 to 14 post fledging. The lines indicate the percentage of responses to playback (pen and paper scoring of attentive behaviour and referring to adults as itemised in the Materials and Methods Section). As the Figure shows, the continuous line shows the increasing reactivity of juveniles to playbacks of neighbours but not to strangers. This reactivity is expressed in vigilance behaviour, in looking towards adults or even walking or running towards adults. Prior to week 8, there were no behavioural responses recorded in juveniles while, by the 12th, week 40 percent of playback yielded a noticeable response, rising to 70 percent by week 14 [48], while the stranger calls created no such response and remained weak or non-existent (at chance level).

The playbacks to juveniles produced no results in vocal responses whatsoever. None of the juveniles emitted any vocalisations in response to any of the playbacks. Importantly, however, non-vocal behaviour was also scored. All adults had shown vigilance behaviour, and if they had been foraging, would stop feeding. For weeks on end of observations of juveniles, however, there were also not even non-vocal responses to the playbacks recorded either. Juveniles initially seemed to be entirely indifferent to these playback vocalisations, be these calls of neighbours and strangers, at least until week 10 post fledging. As said before, they did not show any engagement at all. The exception was the inspection of the speaker and examining it in detail (Figure 10 above). Only after week 10 did post-fledging juveniles start to show some kind of response, not vocally, but by changing behaviour, be this by adopting a vigilance posture or by approaching adults (Figure 14).

Having been unable to obtain any data for juveniles on vocal responses, it was quite clear that the results of this section would at best be preliminary and too small to be statistically evaluated. It was hoped, however, that this first exploration of juvenile vocal development would provide enough information on which to later base a larger fieldwork set of experiments. The results are therefore reported here because they are suggestive of the juvenile development of sensitivity to what is, or might not be, a serious threat to territory. A change in perceptual thresholds feeds into the question of how juveniles learn to distinguish between neighbours and strangers and assess the relative importance of their calls inside the juveniles’ natal territory.

Indeed, in the last weeks of observation (weeks 12–14) any changes in behaviour were in the non-vocal range. A gradual change of juvenile behaviour was noted between weeks 10 and 12 and then quite obviously expressed by week 14. In weeks 12–14, when playback started, juveniles responded by adopting a vigilance posture and occasionally engaging in scanning behaviour. The most pronounced change in behaviour was looking at the adults and/or by walking or running towards the adults as soon as they heard the playback of neighbour calls. As Figure 14 shows, this occurred exclusively when the neighbours’ vocalisations were played back but not when those of strangers were played. As Figure 14 also shows, up to week 8 there were no responses at all, some reactivity (20% of all non-vocal behaviour scored, including foraging and play bouts) was scored by week 10, and within four weeks (week 14), running towards the adults or looking at them jumped to 70 percent of all their displayed behaviour (Figure 14) during playback, meaning they had stopped any pursuits such as foraging, playing or preening once they heard the playback. Instead, their behaviour suddenly focussed on the adults.

## 4. Discussion

Adult magpies are known to have large repertoires of song and a wide variety of calls. With regard to calls, many are directly related to predator detection and territoriality. For instance, to date, at least one of the 27 variations of alarm calls have been found to be referential [45]. Referential signals are stable and universally understood signals (at least by conspecifics) with a specific semantic content. In the case of magpies, we discovered an “eagle” alarm call that warns of the presence of an eagle. When playing back the sound, even when the sound source is on the ground, magpies will look up and scan the sky for birds of prey [46,50,51].

This study was interested in juvenile vocal communication and development and how much of the complex adult repertoire had developed and been used by juveniles prior to their dispersal. Of particular interest were calls for which the biological function of territoriality had been established. Surprisingly, this study found that even at 3 months post fledging, juveniles voiced less than half of the available acoustic elements expressed in calls by the adults. However, an earlier study of juvenile vocal development in magpies [17] had found that song practice begins early at nestling stage and that practising and expanding this repertoire occupies much of the nestlings’ day, but only while parents are absent [17]. Hence, the personal song of a juvenile developed quickly and to a degree even with some virtuosity. However, when it came to calls that form a very central part of the magpie’s communication system, juveniles were silent. While the juveniles expressed affect vocally, none of the copious shorter calls adults emitted (relating to territory or danger) were voiced by juveniles. It is not vocal ability that prevents the expression of calls, but one might well speculate that non-expression is based on a regulatory process. Selectively, those sounds specifically associated with predators and territoriality are not voiced.

Testing the adults yielded strong responses to playbacks within the territory and the responses were differentiated by what the magpies had heard suggesting that there are variations in meaning of these calls. It would certainly appear that warble–carols do not have the same agonistic weight as carols do, and even here, distinctions were made responding with tonal carols or with noisy and harsh ones on the basis of the type of vocalization played back to them. Every one of the three types of playback of the vocalisations of strangers yielded statistically significant increases in the vocalisations of the tested birds. In warble–carols of strangers, and in the carol playback, despite the strong and sharp initial response by the adults on playback, the rate of responding dropped significantly. However, when the warble–carol combination was that of neighbours and it was played back within their territory, the response continued beyond the playback period. Neighbourhoods, to come back to Fisher’s [12] argument, are meant to be stable because of mutual respect for territorial boundaries. When neighbours flaunt such basic rules (which could only happen intentionally), this would present a real danger and create a disequilibrium. As said before, border skirmishes are not uncommon in magpies [3]. Even in this relatively small sample tested here, it emerged that there were clear differences in vocal responses by adults to strangers versus neighbours.

By contrast, the only sounds expressed by juveniles were those concerned with personal well-being and emotions (i.e., anger, fear, surprise, annoyance), usually in the context of the company of other juveniles. Not a single one of them related to territoriality or danger. All the other categories were not expressed but they represent sounds that were also not outside the physiologically vocal reach of juveniles as found in previous investigations [16,31]. Table 2 shows that the vocal categories rarely or never voiced by juveniles, the first five categories, are directly or indirectly connected to territoriality.

This is a significant but also baffling finding in so far as we have evidence for the first time that a songbird with all-year round territory excludes juveniles from expressing anything to do with territoriality or danger. They are permitted, and have been scored to express, individual emotions, as well as likes and dislikes to other juveniles and calls for help from the parents.

As another study has shown [17], juvenile magpies over time show increased competence in delivering longer sequences of song, longer phrasing without breaths, faster rhythms, and smoother sounds, partly explicable by increasing age (associated with lengthening of trachea, beak, neck, and muscles [30]) but not as a result of being tutored. Production techniques, as we investigated [31], increased the acoustic diversity of song but age explained the improvement in performance. In other words, the individual repertoire of song (warble) is already substantial by the time offspring fledge [16] and maturational restraints, perhaps with the exception of carolling, do not apply and cannot explain why vocal expressions were so limited even at a time when the juveniles were semi-independent and competent in foraging by themselves.

To my knowledge, no other studies have specifically reported a similar lack of integration of repertoire in juveniles of a species with large repertoires and all-year round territories.

There would have been few conclusions one could have drawn in terms of learning or development were it not for the chance observations of some repeat social and vocally inappropriate behaviour by juveniles that were punished by adults. The juveniles who disregarded the rule of vocal non-participation in key areas of calls about territoriality and alarm calling seemed to know that their vocal actions had been incorrect because they immediately adopted the most submissive of postures by throwing themselves onto their backs before adults even got to them for a punishing peck. These vocal misdemeanours, observed only in two groups (one in each), provided a window into the non-expression of so many vocalisations, be these concerned with territoriality, predator alarm calls or other territorial alerts. In one case, such as group carolling, some juveniles attempted to use/join in a chorus but were immediately discouraged (Figure 12) and refrained from vocal participation.

Thus, there is evidence that classes of vocalization were treated very differently. Juvenile magpies were obviously allowed to vocally express distress and fear resulting unfailingly in very swift support and protection by the parents. They were allowed to use their begging calls without any restrictions and they were given some free reign to express other emotions even though briefly.

To reiterate a point made in the introduction, Tobias et al. [9] argued that the key factor in the evolution of communal signalling is territoriality; that is, it lies in a combination of long-term territory ownership and social bonds created out of a need to defend valuable ecological resources. If one accepts the premise by Tobias et al. [9], and it is not difficult to do so, then it is more than noteworthy that juveniles expressed less than 40 percent of the call types that were well within their auditory and vocal range and experience.

**Figure 15:**
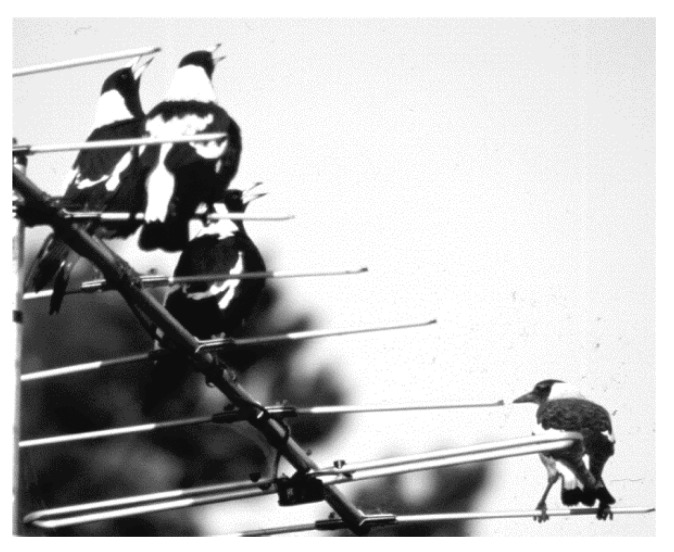
Group carolling with the exclusion of a juvenile (sitting apart and not vocalising) on the extreme right of the image.

The calls not used are important to communicate territorial ownership, raising the question of why especially these signals were omitted. One’s expectations might have been that these calls were going to be perfected and practised well before the juveniles’ dispersal. In reply one might draw the conclusion that calls and territorial ownership go together. So many calls were never used by juveniles precisely because their *natal* territory is not their own *permanent* territory. Generally speaking, they remain guests for the time of growing up and then have to find their own plot in which they can then develop the communication skills in which magpies so clearly excel. However, it leaves the juveniles in a state of tension and conflict. As Figure 15 shows, the single juvenile (two other juveniles did not follow the adults to the television antenna) appears to have wanted to join in with the carolling adults but was left mute and excluded from the group bonding exercise.

Following this line of argument would also lead one to the conclusion that each territory creates its own “identity” based on vocal repertoire. Inexperienced juveniles could send misleading, wrong or weak messages that might confuse neighbours (as well as the parents), lead to false alarms or even neighbour conflict.

In duetting by pairs, so it has been argued, a better fit in the duetting performance may indicate a strong and experienced pair collaborating [3], and this audible display alone may convince some contenders wanting to compete for the territory to give up on such plans. By presenting a strong front, as vocally expressed in various call types (see Figure 9 above), the chances of holding onto a territory may be substantially enhanced. One magpie family I was able to study many years ago held one territory continuously for 18 years, and some families may even succeed in holding a territory over a life-time [26].

Much of this ability to defend a territory for life is due to vocal communication, to a well-coordinated set of actions by at least a bonded pair and to a range of strategies on how to deal with danger. There have been a range of studies that would confirm this. Moseley [52], for instance, showed in swamp sparrows, *Melospiza georgiana*, that high-performance vocal displays were very effective and that opponents responded very differently to strong and weak signals, as was found also in the study of vocal signals in black-throated blue warblers, *Dendroica caerulescens* [53]. Argued in another way, one could speculate that juveniles, if they were to suddenly present a weak and possibly even unformed set of signals, could risk the safety of the territory if such juvenile calls communicated to another group of the same species that they would not be able to withstand an attempt to take over their territory.

Ecologically, this appears logical. Juveniles come and go, and if they interfered in the message system, the entire fragile balance of the relationship with neighbours could be compromised. Conversely, when juveniles eventually form their own territory, their own vocal behaviour would presumably and unambiguously identify them as new territory owners. The results showed at least some evidence, if late in the juvenile development (from week ten onwards), that playbacks within the natal territory increasingly triggered reactivity to calls of magpies that did not belong to their natal group.

In support of the dear enemy hypothesis [54,55], reactivity by juveniles was demonstrated by their response to calls of neighbours but not of strangers. Such reactivity, however, was confined to non-vocal behaviour, confirming that it is important not just to measure vocal responses but other changes of behaviour as well. Juveniles began to display vigilance behaviour (including cessation of any activity in which they had engaged prior to the playback), standing very still with extended neck, then increasingly starting to scan the environment and beginning to seek some reassurance from the adults by looking at them or even running towards them.

The neighbour–stranger discrimination has been discussed in many songbirds but the results in this study suggests a further variant to be considered. Moser-Purdy and Mennill [56] had queried some prior evidence that large song repertoire in a songbird species constrained neighbour–stranger discrimination. They conducted playback experiments with red-eyed vireos, *Vireo olivaceus*, a songbird species with a large song repertoire, and found that repertoire size had no effect on discriminatory abilities. However, responses to strangers were far more aggressive than to neighbours, with lower latency to approach the loudspeaker and using more vocalisations that had already been identified as containing a threat message (often followed by attack).

So why did the 14-week, post-fledging juveniles show stronger, if non-vocal, responses to neighbours rather than to strangers? Had they misunderstood where the risks lie? Moser-Purdy and Mennill [56] also compiled a detailed list of songbird studies showing a capacity to discriminate between neighbours and strangers, so far identifying just 33 species. Of those, only two species showed stronger responses to neighbours than to strangers, and these were the tufted titmouse, *Baeolophus bicolor* [57], and the Eurasian wren, *Troglodytes troglodytes* [58]. The Australian magpie could well be added to this list.

The two main hypotheses concerning neighbour and stranger management that are well-established in the literature make two very contrasting assumptions and findings. The “relative threat” hypothesis [6] rests on the assumption that strangers pose a greater threat to territory holders than do neighbours. The strangers may be non-territorial “floaters” looking to establish a territory by taking over an existing territory. This may well be so in the case of breeding territories, but to reiterate, “territory” does not necessarily mean the same in all species with territorial interests. Most pairs, so far identified and studied, have breeding territories, i.e., territories that are seasonal and may need to be re-established each year anew. All-year round territoriality has not been investigated often, making it possible to argue that there could be important differences in behaviour based on the permanence of the territory.

The results here suggest that in occupied magpie territories, possibly even with defensive neighbourhood alliances, a single stranger has little chance of taking over a territory. However, neighbours, as a group and once they are strong enough in numbers, may well try to expand their own territory or even take over that of their neighbours. Indeed, border skirmishes are not uncommon and have been described in detail elsewhere [3], but most of them are resolved within minutes and only some of them end in small losses of territory to one party when peace is restored.

Hence, the stronger reactivity to calls by neighbours compared to reactivity to that of strangers suggests that the juveniles had distinguished between neighbours and strangers and seemed to be more anxious about neighbourly vocal incursions than those by strangers. It is more difficult to explain the complete lack of responses to a stranger call. One would assume that an unfamiliar auditory event might induce more reactivity, but it did not.

Since the playback unit was placed just 5 m inside the territory (of territories that were at least 8000 m^2^ in size) the reported findings also suggest that, in order to be aware that the calls came from inside their natal territory, the juveniles had to have excellent spatial ability and knowledge of the territorial boundaries.

We know that magpies, despite reaching sexual maturity with the first moult, i.e., in the first year, actually take years to find a territory and usually do not even attempt to breed before they are 5 years old. Secondly, and importantly, we now know that songbirds may learn merely by listening. Evidence of such learning by listening is provided by the occurrence of mimicry even in juvenile magpies. There was ample time and opportunity within their natal territory to have been exposed to a large range of calls.

Five territories (10 adult magpies) produced substantial data for this project. It casts doubt over any assumption that call combinations (warble–carol) were arbitrary signals. It could be tested whether there are territorial calls that not only change their message by introducing noise into the call or whether combinatory calls can carry a less agonistic or more hostile message. This was beyond the scope of this project and seems tempting to test in future.

## 5. Conclusions

Magpies with year-long, and often life-long, territories have a large range of vocal signals concerned with intruders and predators and one main call, carolling, is relatively stereotyped to advertise their territorial ownership. As opposed to species maintaining seasonal territories, it appears that there are very specific rules applied to who can and cannot participate in territorial calls and territorial defence. The data from this set of field experiments suggests that juveniles have no part in contributing acoustically or otherwise to territorial maintenance within their natal territory. Indeed, the results strongly suggest that they can begin to express the range of calls (over half of the vocal signals used by adults) so essential for safeguarding a territory only well after leaving their natal group.

There are several interrelated issues that might be able to explain this phenomenon. The first concerns theories of signal detection. Juveniles may or may not choose to or be able to decipher signals from neighbours or strangers, either because of selective attention or, as described in the literature of psychophysics, as a bias in a subject’s criteria [59].

Listening and memorising become two key elements in non-expressed vocalisations, discussed in detail elsewhere [60]. Once magpies leave their natal territory, they may join bachelor flocks of unattached individuals (in which they may spend several years). In such temporary alliances, group choruses have been observed to engage in choruses, using carolling. One might surmise that a group chorus suggests some commitment to and defence of a coalition/allegiance even within such a temporary framework. We also know that, not unlike tropical mockingbirds, *Mimus gilvus* [61], magpies also seem to be able to distinguish individuals by vocal signals alone, but this will require further testing.

The idea, as proposed here, that some vocalisations may be taboo for some group members, i.e., for offspring in the natal territory, raises many new questions. As a hypothesis, it might be fruitful to examine in the future whether silence may also have a regulatory function in communication among territorial songbirds and such selective processes might well be adaptive.

## Figures and Tables

**Table 1:** Post-fledging sound production development. Call classifications presented in same order as in Figure 9 above. N: The category “never”; O: “Occasional”, included any sounds recorded to be produced by juveniles twice per month and up to once per week. R: “Regular”, meant voicing specific sounds at least once per week or more.

Call Classification	Weeks 1–4 Month 1	Weeks 5–8 Month 2	Weeks 9–12 Month 3	Weeks 13–14 Month 4	Overall Occurrence
1. Generic alarm call	N	N	O	O	Sibling-specific
2. Generic noisy alarm	N	N	N	O	Situational/surprise
3. Mobbing call noisy	N	N	N	N	N
4. Mobbing call tonal	N	N	N	N	N
5. Anger (noisy)	N	N	N	N	N
6. Group carolling	N	N	N	N	N
7. Disapproval	N	N	N	N	N
8. Fledgling Begging	R	R	R	O	Decreasing
9. Coo	N	N	N	N	N
10. Harsh alarm calls (a–f)	N	N	N	N	N
11. Anger (tonal)	N	N	O	O	Sibling-specific
12. Eagle alarm call	N	N	N	O	
13. Group carolling	N	N	N	N	N
14. Individual carolling	N	N	N	N	N
15. Highest alert/alarm	N	N	N	N	N
16. Highest alert/variations	N	N	N	N	N
17. Fear call	O	O	O	O	Facing parental rebuke
18. Food call	N	N	N	N	N
19. Song (warble)	R	R	R	R	When alone
20. High alert/high-pitched	N	N	N	N	N
21. High danger	N	N	N	N	N
22. Distress	N	N	O	O	S = Sibling play fights
23. Aerial dispute	N	N	N	O	S = with neighbouring juvenile
24. High distress	N	O	N	N	Caught by goshawk
25. Extreme fear	N	N	N	O	Caught by goshawk
26. Mimicry	N	N	O	O	When alone

**Table 2:** Functional clusters in social and territorial communication. The table shows the call types that juveniles did *not* express (in the shaded areas (I–V). The calls they did express in VI have no shading. A cross-reference is provided between acoustic characteristics with function (using the numbering of Table 1).

I Notifying Others	II Issuing Warnings	III Calls To Arms	IV Expressing Territorial Coalitions	V Controlling/Issuing Behaviour	VI Expressing Emotions Directed as Others
high alert or high danger calls (10, 20, 21)	noisy, harsh or highest alert alarm calling (2, 10, 15, 16, 21)	Mobbing Calls (3, 4)	group or individual carolling (13, 14)	coo, or food calls (9, 18)	Disapproval (7), Anger (5)
